# The potential benefits of “community champions” in the healthcare system

**DOI:** 10.1177/08404704231179911

**Published:** 2023-06-02

**Authors:** Aisha Lofters, Vijayshree Prakash, Kimberly Devotta, Mandana Vahabi

**Affiliations:** 1 7985 Women’s College Hospital, Toronto, Ontario, Canada.; 2 University of Toronto, Toronto, Ontario, Canada.; 3 St. Michael’s Hospital, Toronto, Ontario, Canada.; 4 Toronto Metropolitan University (formerly Ryerson University), Toronto, Ontario, Canada.

## Abstract

In a study to understand acceptability and uptake of Human Papilloma Virus (HPV) self-sampling, we engaged community champions to lead recruitment and other study activities. This article describes qualitative findings relevant to the role of the community champion. We found that community champions were critical to promoting awareness about and encouraging cervical screening and HPV self-sampling. They were well-connected community members who had healthcare backgrounds, which created trust in their messages. They were highly effective at encouraging screening because of their education and cultural congruency, combined with the time for thorough and clear explanations. Women had an inherent level of comfort with the community champions that often did not exist with their physician. The community champions were seen as being able to address some of the barriers that exist within the healthcare system. We encourage health leaders to consider how this role can be sustainably and meaningfully incorporated into the healthcare system.

## Introduction

Cervical cancer is a relatively unique cancer in that it is almost exclusively caused by the Human Papilloma Virus (HPV) and in that it has the potential to be eliminated through regular screening and vaccination.^1,2^ Cervical cancer is highly reflective of health inequities.^1^ In Canada, despite a universal healthcare system where screening comes at no cost to citizens and permanent residents, cervical screening uptake is consistently lower among women who are foreign-born and/or living with low income.^3-9^ Women who have immigrated from South Asian, Middle Eastern, and North African countries have been found to have particularly low cervical screening uptake, and potentially a higher incidence of later stage cervical cancer.^9-12^ Health leaders and policy-makers need new strategies to reach these groups, and others facing structural marginalization, for cervical screening.

Self-sampling for HPV has shown promise as a method to increase cervical screening.^13-24^ We recently conducted a multi-method study in Ontario, Canada, to understand the acceptability and uptake of a take-home HPV self-sampling kit among women who self-identified as South Asian, West Asian, Middle Eastern, and North African.^13,25^ Women were offered the chance to try HPV self-sampling, and both those who did and did not try the kit provided feedback through survey and telephone follow-up. We also invited women to participate in focus groups and key informants to participate in semi-structured interviews to further understand facilitators and barriers to screening.

Community champions (also known as community health workers, community ambassadors, and peer workers) have been found to be key to reaching groups deemed as “hard-to-reach” and have been used with Lto educate on cancer screening and increase cancer screening uptake.^6,26-30^ Thus, we engaged community champions to lead recruitment and other study activities. Community champions were trusted women in the communities with healthcare backgrounds from their home countries who had pre-existing social and organizational connections. This article specifically describes qualitative findings relevant to the role of the community champion in encouraging study participation and cervical screening uptake.

## Methods

The methods for this study have been described in detail elsewhere.^13,25^ Briefly, we used a community-based mixed methods design. Women eligible for inclusion were aged 30-69 years, eligible for cervical screening (i.e. had ever been sexually active and no history of hysterectomy), self-identified as South Asian, West Asian, Middle Eastern, or North African, lived in the Greater Toronto Area of Ontario, Canada, reported at least four years since their last Pap test including no history of Pap test, and were able to communicate and provide consent in English. We excluded women who were pregnant as the HPV self-sampling device to be used in the study had not been approved for use on pregnant women.

Over the life of the study, a total of three community champions recruited participants through locations and events such as community organizations, places of worship, cultural events, entertainment events, parenting groups and local tea parties, and using techniques such as distributing flyers, formal group presentations, and informal conversations. All participants were given the option of trying the HPV self-sampling kit, but this was not a criterion for study participation. All participants, regardless of their choice, completed an interviewer-administered survey about their cervical cancer knowledge, attitudes, and practices and were given printed information on cervical screening and a $30 honorarium. While being able to converse in English was a requirement of study participants, community champions were fluent in multiple languages to be able to explain or interpret difficult concepts. Community champions successfully recruited 108 study participants.^13^ Subsequently, three focus groups were held with women who had chosen to use the kit and two with women who had not to further explore barriers and facilitators to HPV self-sampling and cervical screening, including using community champions for recruitment. The focus groups ranged from four to eight participants, with a total of 29 women across all five groups. Women were again compensated a $30 honorarium for focus group participation. We also held eight semi-structured telephone interviews with key informants, that is, the community champions and leaders of groups from whom we recruited participants to understand their perspectives on implementation barriers and facilitators for HPV self-sampling and cervical screening.

All focus groups and interviews were audio-recorded and transcribed verbatim. Transcripts were analyzed using inductive thematic analysis with a reciprocal coding approach.^31^ Two authors created the initial codebook using the same subset of transcripts, and then met with the other two authors to discuss the interpretation and codes. After the initial codebook was confirmed by the team, one author coded the rest of the transcripts. An additional team meeting was held to discuss the final list of codes and review the coding. We used NVivo version 12 for data management and analysis. Here, we describe previously unpublished themes that emerged from focus groups and interviews specific to the role of the community champion.

## Results

Key themes that emerged from interviews and focus groups relevant to the role of the community champion in encouraging study participation and cervical screening uptake included the role description of the community champions, the effectiveness of the community champions in encouraging screening, and the system gaps that could potentially be addressed by the community champions.

### The role description of the community champions

Key informants described the characteristics and qualifications needed for the community champion role. The community champion role was viewed as a highly supportive one, where the champions were pre-existing members of the community who were knowledgeable about health (all community champions had been healthcare professionals in their home countries although not practicing as such in Canada), trustworthy, connected, and could communicate in languages in which community members felt comfortable. One of the community champions described the key elements of the role thus:

… So, my health background plus my knowledge of the existing scenario here and me being their friend so all three factors made them talk to me about screening ... For most women it’s the personal connection that matters ... being from the same background makes me understand their family situation and also their approach towards screening … [Key informant, interview 2]

She described her activities as a community champion, both in this project and other cancer screening roles, as one of being available in different settings where people felt comfortable and providing information in a variety of ways:

… I went out to the community and places of worship, different community centres, at newcomer centres, at the libraries so like the information was easy, available to people by means of brochures, by means of group discussions, events, cultural festivals, booths and all possible outreach means. [Key informant, interview 2]

This approach of being embedded in the community meant that the champions were able to interact with women in spaces where they felt safe and comfortable:

… we have some women’s circle so in the, in this circle we are talking about … any issues … in that, you know the friendly atmosphere and they feel comfortable over there with the open air … sometime husband issue sometimes you know the children issue or just sometimes lots of thing you know when you are settling down in new country … [Key informant, interview 4]

Key informants noted that the community champions were the “face” of the project in the community, and that their amiability was a critical component of their role:

… actually, I had met her for another project before … a person like [community champion] who you know [pause] the face for this project in our community she was very personable … [Key informant, interview 8]

… she [community champion] had interaction with our client as well. She used to come to our organization …… not to … interrupt the classes … she was having a desk at a common place and she had very good interactions with the people and like usually providing them information. [Key informant, interview 6]

The ability to speak to women in their home languages was also highlighted by key informants:

… females they prefer female and their own language. This is a barrier ... [Key informant, interview 4]

… the main barrier we found was the language … if [pause] you can speak the same language you’re able to kind of really convince about the different aspects of the test … if there is someone who is like [community champion] is able to [pause] tell them all … [Key informant, interview 7]

### The effectiveness of the community champions in encouraging screening

Focus group participants felt that the community champions were highly effective in encouraging screening, and this effectiveness seemed to be due to the characteristics described above by key informants. The champions were able to take the time (often over multiple conversations) to provide clear and thorough explanations, which healthcare providers often did not do. These clear explanations in turn built confidence in women that they would be able to undertake self-sampling:

…and the best part is…[community champion] explained …., in very friendly manner…and I understand the whole steps and everything. [Focus group A, participant 4]

…that [community champion] explained things so well and make it so simple and easy. I think we need more consultants like that who will help the people than really going through the doctor, clinic, standing in the queue, all this big process and then wait three weeks to understand everything is okay or come back again with I think we have a problem so maybe we need to improve this process as well. [Focus group B, participant 6]

The community champions were in a unique position as they had healthcare backgrounds, which added credibility to the information and education they were providing to participants:

… you know so [community champion] is a doctor. She, I know that I can talk to her ... [Focus group C, participant 2]

.. like I knew [community champion] so I was, I knew whatever she is giving me, she is a reliable source. [Focus group D, participant 2]

### System gaps that could potentially be addressed by community champions

Community champions were seen as being as one potential approach to help address current gaps and barriers in the healthcare system. For example, women expressed a level of discomfort with their own primary care physicians, which they did not have with the community champions:

I was aware of cervical cancer but when [community champion] approached me you know it's like for me going to doctor is not a very pleasant thing because I've never been very comfortable going to the doctor's …-but the way - [community champions] are approaching everyone and educating everyone it is actually a very good thing and I hope these, because many of the women they have fear of going to doctors for all these personal tests … [Focus group D, participant 4]

… one of the barriers that came up by our, during all of my work and through studies was that women did not want to go to the doctor … hesitant, not only going to the male doctor but even going to the doctor was one extra step for them... so their doctor is from the healthcare background but they wouldn't talk to the doctor because for them it's that personal connection that matters. For most women it's the personal connection that matters. [Key informant, interview 2]

… but for my cousin it was a huge problem because she's in new country, she feels shy to go to male doctor ... So, [community champion] encourage her with many explanation and clear explanation to use it and she take a step to do it by herself. I was really proud of her because it was so easy and it was as a first experience with the medical things … in Canada and she got some, it's called like a positive experience. [Focus group B, participant 5]

Women also expressed that there were unmet information needs about cervical screening in their communities, sometimes due to how busy primary care physicians are. The community champions were seen as potentially able to meet these needs:

I want to add that, with a doctor they don't have time to explain ... I cannot blame doctor[s]. They are all very busy … [Focus group A, participant 3]

We don't get much information … on these things and we generally don't talk amongst ourselves also about this so when this study came out that was an opportunity where we could discuss about cervical cancer and about the ways and means for screening. [Focus group A, participant 7]

… because ….. we don't know, about it [cervical screening] you know why it is important but when you know [community champion] explain and you know the, those kind of things so then I realize oh, how is, this is important for us and the ladies so I think so it's more on, more knowledge is more effective and people they can take step. [Focus group A, participant 3]

I think peer communication is very important and influential but like with all behaviour change communication you have to have that message reinforced in multiple ways you know so you have to hit your target audience with peer education, with advertisements, social media …. but I personally agree that if my friend for example if [community champion] tells me about something I'm more likely to pay attention because I would think that she has already screened the information, verified it and is then only giving it to me so somebody I trusted, a source for me would be somebody who I know has expertise in that area and is able to give me trusted information. [Focus group C, participant 3]

## Discussion

In this community-based study, we found that community champions were critical to our recruitment success and thus critical to promoting awareness about and encouraging cervical screening and HPV self-sampling. Community champions were well-respected, well-liked, and well-connected members of the relevant ethnocultural communities who had healthcare backgrounds, which created or increased community members’ trust in their messages. They met women where they were, and they interacted with women in a variety of safe community spaces and in a variety of ways. They were highly effective at encouraging screening and HPV self-sampling because of their health education and cultural congruency combined with having the time for thorough and clear explanations, often over multiple conversations and over a period of time, which many primary care physicians did not provide. Women also had an inherent level of comfort with the community champions that often did not exist with their primary care physician. As a result, the community champions were seen as potentially being able to address some of the barriers that exist within the healthcare system for women from these ethnocultural groups. Because of their healthcare backgrounds, they were able to initiate engaging conversations about health, outside of healthcare spaces.

Our findings highlight the key role that community champions can play in encouraging preventive care such as cervical screening for communities where the prevalence of under-screening and informational needs are high and who may experience systemic barriers to care. Community champions were successful at encouraging screening and giving women the confidence to self-sample. Meaningful empowerment-based health teaching has previously been noted to require respect for culture, a sense of community, the time and space to identify barriers and facilitators, and tellingly the use of lay leaders.^32^ Our community champions appeared able to empower women through these principles.

Inequalities in cervical cancer screening in Ontario for immigrant women have been extensively documented, and do not appear to have improved over time.^3-6,11,33^ New strategies are needed to reduce inequities, and healthcare leaders must recognize that these strategies may need to be community-based instead of, or alongside, strategies that are primary care-based. De Alba et al. previously found that women who do not speak English are less likely to have their primary care provider recommend cervical screening,^34^ and our previous work showed that only 77.7% of Ontario women surveyed listed their family physician as a source of health information.^35^ This number fell to 69.2% for women classified as “family first,” that is, those who tended to put their family’s health ahead of their own, more than 20% of whom were South Asian-born.^35^ The need to look outside of primary care has become even more urgent for healthcare leaders in recent times, as leaders also must acknowledge that the primary care system is currently under significant strain coming out of the COVID-19 pandemic.^36^

However, integrating community champions into the system will come with challenges. A concrete role definition, required qualifications, clinical boundaries, liability, high quality training, clear referral pathways to and from the healthcare system, how to spread and scale, and sustained funding are just some of the elements that would need to be carefully thought through. It is also not clear if community champions will be able to support those often deemed “hardest to reach” such as those who have no English language ability, are underhoused, and are actively suffering from addictions. Meaningfully incorporating community champions into the healthcare system must be done in a way that allows for continuous evaluation, quality improvement, and long-term sustainability (including community champion compensation). Canada has been called a land of “perpetual pilot projects” where community-based projects are easily jettisoned,^37^ so a challenge that health leaders are now faced with is, for which there are unfortunately no quick and easy solutions, how to approach the use of community champions to address health inequities in a manner that does not fall into this same trap. In the COVID-19 pandemic, the city of Toronto alone was able to mobilize over 720 community ambassadors, reflective of the cultural, language, and racial diversity of the city, to engage community members on COVID-19 vaccination.^38^ A program evaluation found that these ambassadors increased vaccine confidence, access, and update, and reduced barriers to screening.^38^ Building on and expanding from the community structures and healthcare-community partnerships used in the pandemic could lead to meaningful and sustained use of community champions throughout the healthcare system.

## Conclusion

One way that inequities in cervical screening, and other types of preventive care, for particular ethnocultural communities may be able to be addressed is through the use of community champions—trusted members of the community, perhaps with healthcare backgrounds, who are able to educate and support in safe settings and in languages in which community members feel comfortable. We encourage health leaders to consider how this role can be sustainably and meaningfully incorporated into the healthcare system.
